# Proper conditional analysis in the presence of missing data: Application to large scale meta-analysis of tobacco use phenotypes

**DOI:** 10.1371/journal.pgen.1007452

**Published:** 2018-07-17

**Authors:** Yu Jiang, Sai Chen, Daniel McGuire, Fang Chen, Mengzhen Liu, William G. Iacono, John K. Hewitt, John E. Hokanson, Kenneth Krauter, Markku Laakso, Kevin W. Li, Sharon M. Lutz, Matthew McGue, Anita Pandit, Gregory J. M. Zajac, Michael Boehnke, Goncalo R. Abecasis, Scott I. Vrieze, Xiaowei Zhan, Bibo Jiang, Dajiang J. Liu

**Affiliations:** 1 Department of Public Health Sciences, Penn State College of Medicine, Hershey, Pennsylvania, United States of America; 2 Center of Statistical Genetics, Department of Biostatistics, University of Michigan, Ann Arbor, Michigan, United States of America; 3 Department of Psychology, University of Minnesota, Minneapolis, Minnesota, United States of America; 4 Institute for Behavioral Genetics, University of Colorado Boulder, Boulder, Colorado, United States of America; 5 Department of Epidemiology, Colorado School of Public Health, University of Colorado Anschutz Medical Campus, Aurora, Colorado, United States of America; 6 Institute of Clinical Medicine, Internal Medicine, University of Eastern Finland and Kuopio University Hospital, Kuopio, Finland; 7 Department of Biostatistics and Informatics, University of Colorado, Anschutz Medical Campus, Aurora, Colorado, United States of America; 8 Department of Clinical Science, Quantitative Biomedical Research Center, University of Texas Southwestern Medical Center, Dallas, Texas, United States of America; Newcastle University, UNITED KINGDOM

## Abstract

Meta-analysis of genetic association studies increases sample size and the power for mapping complex traits. Existing methods are mostly developed for datasets without missing values, i.e. the summary association statistics are measured for all variants in contributing studies. In practice, genotype imputation is not always effective. This may be the case when targeted genotyping/sequencing assays are used or when the un-typed genetic variant is rare. Therefore, contributed summary statistics often contain missing values. Existing methods for imputing missing summary association statistics and using imputed values in meta-analysis, approximate conditional analysis, or simple strategies such as complete case analysis all have theoretical limitations. Applying these approaches can bias genetic effect estimates and lead to seriously inflated type-I or type-II errors in conditional analysis, which is a critical tool for identifying independently associated variants. To address this challenge and complement imputation methods, we developed a method to combine summary statistics across participating studies and consistently estimate joint effects, even when the contributed summary statistics contain large amounts of missing values. Based on this estimator, we proposed a score statistic called PCBS (partial correlation based score statistic) for conditional analysis of single-variant and gene-level associations. Through extensive analysis of simulated and real data, we showed that the new method produces well-calibrated type-I errors and is substantially more powerful than existing approaches. We applied the proposed approach to one of the largest meta-analyses to date for the cigarettes-per-day phenotype. Using the new method, we identified multiple novel independently associated variants at known loci for tobacco use, which were otherwise missed by alternative methods. Together, the phenotypic variance explained by these variants was 1.1%, improving that of previously reported associations by 71%. These findings illustrate the extent of locus allelic heterogeneity and can help pinpoint causal variants.

## Author summary

It is of great interest to estimate the joint effects of multiple variants from large scale meta-analyses, in order to fine-map causal variants and understand the genetic architecture for complex traits. The summary association statistics from participating studies in a meta-analysis often contain missing values at some variant sites, as the imputation methods may not work well and the variants with low imputation quality will be filtered out. Missingness is especially likely when the underlying genetic variant is rare or the participating studies use targeted genotyping array that is not suitable for imputation. Existing methods for conditional meta-analysis do not properly handle missing data, and can incorrectly estimate correlations between score statistics. As a result, they can produce highly inflated type-I errors for conditional analysis, which will result in overestimated phenotypic variance explained and incorrect identification of causal variants. We systematically evaluated this bias and proposed a novel partial correlation based score statistic. The new statistic has valid type-I errors for conditional analysis and much higher power than the existing methods, even when the contributed summary statistics contain a large fraction of missing values. We expect this method to be highly useful in the sequencing age for complex trait genetics.

## Introduction

Meta-analysis has become a critical tool for genetic association studies in human genetics. Meta-analysis increases sample sizes, empowers association studies, and has led to many exciting discoveries in the past decade [1–5]. Many of these genetic discoveries have informed new biology, provided novel clinical insights [6, 7], and led to novel therapeutic drug targets [8, 9]. Conditional meta-analysis has been a key component for these studies, which is useful to distinguish novel association signals from shadows of known association signals and to pinpoint causal variants.

Existing methods for conditional meta-analysis were proposed based upon the assumptions that summary association statistics from all variant sites are measured and shared. Yet, in practice, the score statistics from contributing studies often contain missing values, possibly due to the use of different genotyping arrays, sequencing capture assays, or quality control filters by each participating cohort. While genotype imputation is an effective approach to fill in missing genotype data for participating cohorts, many scenarios may preclude accurate genotype imputation. For example, a targeted genotyping array/sequencing assay (e.g. exome array) may not provide sufficient genome-wide coverage for imputation. In addition, it is challenging to impute low frequency variants even with the highest quality reference panels. Imputed genotypes of low quality are often filtered out based upon the recommendations from the best practices [10], since these variants are more prone to artefacts and can lead to inflated type I errors. Therefore, missing data in meta-analysis of genetic association studies are unavoidable. Some existing meta-analysis strategies can be highly biased in the presence of missing data.

First, a commonly used method for conditional analysis, COJO, can lead to biased results when contributed summary association statistics from participating studies contain missing values [11]. The COJO method approximates the variance-covariance matrix between association statistics with the linkage disequilibrium (LD) information from a reference panel. When the association statistics from contributed studies are missing at some variant sites, the correlation matrix of the meta-analysis statistics can differ greatly from the LD matrix. Consider the simple example of a meta-analysis of two independent studies, where variant 1 is only measured in study 1 and variant 2 is only measured in study 2. The meta-analysis association statistics for the two variants are independent, which cannot be approximated by the LD. COJO only uses meta-analysis results as input. Therefore, it cannot distinguish the scenario where only study 1 measures both variants (and study 2 measures none), and the scenario where study 1 only measures variant 1 and study 2 only measures variant 2. In the presence of missing data, COJO can be highly biased and lead to inflated type I errors.

Second, the strategy of imputing missing data from contributed association statistics and using imputed association statistics in meta-analysis can also lead to inflated type I errors in conditional analysis. A simple imputation strategy for marginal (or unconditional) analysis is to replace missing summary statistics with zeros (REPLACE0), which are their expected value under the null hypothesis [2, 3]. This method yields valid type I errors for marginal association analysis. Taking this simple approach for conditional analysis, however, is problematic. The genetic variants at conditioned sites are likely to have non-zero effects. Replacing missing summary data with zeros will bias the genetic effect estimates at conditioned variant sites, and can lead to highly inflated type I errors for conditional analysis (see RESULTS). Similarly, the methods that seek to impute missing summary statistics based upon LD (e.g. impG [12]) may introduce substantial biases to the effects of missing variants. Plugging in the imputed Z-score statistics into conditional analysis (impG+meta) can lead to inflated type I errors. Finally, discarding studies with missing summary statistics (DISCARD, or complete case analysis) will give valid type I errors, but at the cost of reduced power.

In the statistics literature, synthesis methods have previously been developed to meta-analyze joint effects from different studies, where the participating studies measure different predictors [13, 14]. The scenario is similar to the meta-analysis of genetic association studies with missing data. Yet, in genetic association analysis, usually only marginal effects are reported and joint effects have to be approximated from marginal effects. The synthesis methods also lack an implementation for genetic association studies, which greatly limits their impact. To explore the usefulness of synthesis methods, we proposed and implemented an extension of the synthesis methods termed SYN+, which can be applied in genetic association meta-analysis.

To overcome these limitations of existing GWAS meta-analysis methods and improve power, we developed an improved conditional meta-analysis method called partial correlation based score statistic (PCBS) that borrows strength across multiple participating studies and consistently estimates the partial variance-covariance matrices between genotypes and phenotypes. We conducted extensive simulations, and showed that our PCBS method has valid type I error and the highest power among all the methods. On the other hand, COJO, impG+meta and REPLACE0 can lead to highly inflated type I errors in the presence of missing data. SYN+, while having valid type I errors, is consistently less powerful than PCBS, especially when the missingness is high or the conditioned variants have larger effects. We also demonstrated the clear advantage of PCBS in the meta-analysis of cigarettes per day phenotype. PCBS identified many more independently associated variants from known loci, compared to alternative approaches.

We implemented the proposed methods in the open-source software tools RAREMETAL [15] and R package rareMETALS and made them publically available (https://genome.sph.umich.edu/wiki/Rare_Variant_Analysis_and_Meta-Analysis). RAREMETAL and rareMETALS use marginal score statistics and exact variance-covariance matrix as input, which is suitable for rare variant association analysis. We also implemented the same method in rareGWAMA (https://github.com/dajiangliu/rareGWAMA), which conducts meta-analysis using approximate covariance matrix from a reference panel. These methods and tools have been applied and tested in a few large scale meta-analyses. We expect these methods to play an important role in sequence-based genetic studies and lead to important genetic discoveries.

## Materials and methods

In this section, we first review the standard meta-analysis methods for single variant and gene-level association tests when analyzing datasets without missing summary statistics from contributing studies. We then illustrated the limitations of the existing methods and described the new method PCBS for valid and powerful conditional analysis in the presence of missing summary statistics from contributing studies.

### Overview of meta-analysis methods

We denote the genotype for individual *i* at variant site *j* in study *k* as *G*_*ijk*_, which can take values of 0,1 or 2, representing the number of the minor (or alternative) alleles in the locus. When the genotypes are imputed or generated from low pass sequencing studies, genotype dosage can be used in association analysis. In this case, *G*_*ijk*_ will be the expected number of minor (or alternative) allele counts. We denote the non-genotype covariates as **Z**_**ik**_, which includes a vector of 1’s to incorporate the intercept in the model. Single variant association can be analyzed in a regression model: **Y**_**k**_ = **G**_**jk**_*β*_*j*_ + **Z**_**k**_**γ**_**k**_ + **e**_**k**_. The score statistic for single variant association takes the form:

$$U_{jk}=\frac{1}{{\hat{\sigma }}_{0}^{2}}\sum_{i}G_{ijk}(Y_{ik}-{\hat{y}}_{ik})$$ (1)

where ${\hat{y}}_{ik}=Z_{ik}{\hat{\gamma }}_{k},\;{\hat{\gamma }}_{k}$ is the covariate effect, and ${\hat{\sigma }}_{0}$ is the standard deviation of the phenotype residuals estimated under the null model M_0_

$$Y_{k}=Z_{k}\gamma_{k}+e_{k},\;e_{k}∼MVN(0,{\hat{\sigma }}_{0}^{2}I)$$ (M0)

Without the loss of generality, we assume that the phenotype residuals are standardized in each study as in commonly done in practice. So ${\hat{\sigma }}_{0}$ is often equal 1 in practice. We denote the vector of score statistics in a genetic region as **U**_**k**_ = (*U*_1*k*_,…,*U*_*Jk*_). The variance-covariance matrix between scores statistics is equal to

$$V_{k}=1/{\hat{\sigma }}_{0}^{2}[G_{k}^{\prime }G_{k}-G_{k}^{T}Z_{k}{\left(Z_{k}^{T}Z_{k}\right)}^{-1}Z_{k}^{T}G_{k}]$$ (2)

For our illustration of the method, we focus on the analysis of continuous outcomes. Yet, the meta-analysis and conditional meta-analysis methods work for both continuous outcomes and binary outcomes.

The meta-analysis score statistics and their covariance matrices are calculated using the Mantel-Haenszel method, i.e. **U** = ∑_*k*_ **U**_**k**_ and **V** = ∑_*k*_ **V**_**k**_. The meta-analysis statistics can be used to estimate the joint effects for variants 1,…,*J*, i.e. $\hat{\beta }=V^{-1}U$.

We denote the score statistics at candidate and conditioned variant sites as $U=(U_{G},U_{G^{*}})$, where **G** and G*represent the genotypes from the candidate and conditioned variants respectively. The variance covariance matrix for **U** equals to $V=\left(\begin{array}{cc} V_{G} & V_{GG^{*}}\\ V_{G^{*}G} & V_{G^{*}} \end{array}\right)$

The conditional score statistic can be calculated by

$$U_{G|G^{*}}=(U_{G}-V_{GG^{*}}V_{G^{*}}^{-1}U_{G^{*}}){\hat{\sigma }}_{0}^{2}/{\hat{\sigma }}_{c}^{2}$$ (3)

where ${\hat{\sigma }}_{c}^{2}$ is the residual variance estimated from the conditional analysis model

$$Y_{k}=G_{k}^{*}\beta_{G^{*}}+Z_{k}\gamma_{k}+e_{k},e_{k}∼MVN(0,{\hat{\sigma }}_{c}^{2}I)$$ (Mc)

After conditioning on the genotypes G*, the residual variance equals to ${\hat{\sigma }}_{c}^{2}={\hat{\sigma }}_{0}^{2}(1-\frac{1}{N}U_{G^{*}}^{\prime }V_{G^{*}}^{-1}U_{G^{*}})$.

It is easy to verify that the variance of the conditional score statistics under M_c_ is equal to

$$V_{G|G^{*}}=(V_{G}-V_{GG^{*}}V_{G^{*}}^{-1}V_{G^{*}G}){\hat{\sigma }}_{0}^{2}/{\hat{\sigma }}_{c}^{2}$$ (4)

The single variant and gene-level tests in conditional analysis can be calculated based upon the conditional score statistics $U_{G|G^{*}}$ and the covariance matrix $V_{G|G^{*}}$. Details are provided in **S1 Text**.

### Partial correlation based score statistics (PCBS)

Reviewing formulae (3) and (4), we note that the conditional score statistics and their variances only depend on the partial variance-covariance matrix between the phenotypes and the genotypes after the adjustment of covariates. The key idea underlying our approach is to derive a consistent estimator for the partial covariances in the presence of missing summary statistics and to use it for unbiased conditional analysis.

In statistics, to calculate the partial covariance between random variables *G*_*jk*_ and *Y*_*k*_ adjusting for variable *Z*_*k*_, we first regress out covariate *Z*_*k*_ from both *G*_*jk*_ and *Y*_*k*_, and then calculate the covariance between the residuals. Specifically,

$${\hat{\rho }}_{G_{jk}Y_{k}|Z_{k}}=\frac{1}{N_{jk}{\hat{\sigma }}_{0}^{2}}G_{jk}^{\prime }(Y_{k}-Z_{k}\hat{\gamma })$$ (5)

For a given study, it is easy to check that the partial covariances are in fact scaled score statistics, i.e.

$${\hat{\rho }}_{G_{jk}Y_{k}|Z_{k}}=\frac{1}{N_{jk}}U_{jk}$$ (6)

$${\hat{\rho }}_{G_{j_{1}k}G_{j_{2}k}|Z_{k}}=\frac{1}{N_{jk}}V_{j_{1}j_{2}k}$$ (7)

Therefore, in meta-analysis, we propose to estimate the partial covariance between genotype *G*_*ij*_, phenotype *Y*_*i*_ after adjusting the covariate effect *Z*_*i*_ using all available summary statistics:

$${\hat{\rho }}_{GY|Z,j}=\frac{\sum_{k\in \{k:M_{jk}=1\}}U_{jk}}{\sum_{k\in \{k:M_{jk}=1\}}N_{jk}}$$ (8)

$${\hat{\rho }}_{GG|Z,j_{1}j_{2}}=\frac{\sum_{k\in \{k:M_{j_{1}k}=M_{j_{2}k}=1\}}V_{j_{1}j_{2}k}}{\sum_{k\in \{k:M_{j_{1}k}=M_{j_{2}k}=1\}}N_{jk}}$$ (9)

Here *M*_*jk*_ is an indicator variable that takes the value of 1 when the summary statistic at variant site *j* is measured in study *k*. For notational convenience, we define the matrices of partial covariance as ${\hat{\rho }}_{GY|Z}={\left({\hat{\rho }}_{GY,j}\right)}_{j=1,\ldots ,J}$ and ${\hat{\rho }}_{GG|Z}={\left({\hat{\rho }}_{GG|Z,j_{1}j_{2}}\right)}_{j_{1},j_{2}=1,\ldots ,J}$. Under the fixed effect model, we have $E(V_{k}^{-1}U_{k})=\beta$ for all *k*. We showed in **S1 Text** that $E({\hat{\rho }}_{GG|Z}^{-1}{\hat{\rho }}_{GY|Z})=\beta$. Therefore, the partial covariance matrices can be consistently estimated even in the presence of missing summary statistics.

We define partial correlation based score statistics as

$${\tilde{U}}_{G|G^{*}}={\hat{\rho }}_{GY|Z}-{\hat{\rho }}_{GG^{*}|Z}{\hat{\rho }}_{G^{*}G^{*}|Z}^{-1}{\hat{\rho }}_{G^{*}Y|Z}$$ (10)

The covariances for ${\tilde{U}}_{G|G^{*}}$ are equal to

$${\tilde{V}}_{G|G^{*}}=cov({\hat{\rho }}_{GY|Z})+{\hat{\rho }}_{GG^{*}|Z}{\hat{\rho }}_{G^{*}G^{*}|Z}^{-1}cov({\hat{\rho }}_{G^{*}Y|Z}){\hat{\rho }}_{G^{*}G^{*}|Z}^{-1}{\hat{\rho }}_{G^{*}G|Z}-{\hat{\rho }}_{GG^{*}|Z}{\hat{\rho }}_{G^{*}G^{*}|Z}^{-1}cov({\hat{\rho }}_{G^{*}Y|Z},{\hat{\rho }}_{GY|Z})-cov({\hat{\rho }}_{GY|Z},{\hat{\rho }}_{G^{*}Y|Z}){\hat{\rho }}_{G^{*}G^{*}|Z}^{-1}{\hat{\rho }}_{G^{*}G|Z}$$ (11)

It is easy to verify that the conditional analysis using the estimator ${\tilde{U}}_{G|G^{*}}$ is equivalent to the standard score statistics when no missing data are present. In the presence of missing data, the partial correlation based statistic ${\tilde{U}}_{G|G^{*}}$ remains consistent. The conditional association analysis can be performed by replacing the standard score statistic with a partial correlation based score statistic. Details for calculating single variant and gene-level conditional association statistics can be found in **S1 Text**.

#### Extensions of PCBS to approximate conditional analysis

For rare variant association meta-analysis, it is recommended to use exact covariance matrix for conditional analysis and for gene-level association analysis. Using a reference panel to approximate the covariance between association statistics may lead to biases, as shown in Hu et al [16]. Nonetheless, our proposed conditional analysis method can also work with approximate covariance matrix for more common variants using LD information from a reference panel. Specifically the covariance between score statistics $U_{j_{1}k}$ and $U_{j_{2}k}$ can be approximated by $cov(U_{j_{1}k},U_{j_{2}k})\approx r_{j_{1}j_{2}}\sqrt{V_{j_{1}j_{1}k}V_{j_{2}j_{2}k}}$, where $r_{j_{1}j_{2}}$ is the correlation coefficient between the genotypes of variants *j*_1_ and *j*_2_ estimated from a reference panel So the approximate covariance matrix for the *k*^*th*^ study can be written as

$${\tilde{V}}_{k}=diag\left(\sqrt{V_{1,1,k}},\ldots ,\sqrt{V_{J,J,k}}\right)Rdiag\left(\sqrt{V_{1,1,k}},\ldots ,\sqrt{V_{J,J,k}}\right)\;\mathrm{with}\;R={\left(r_{j_{1}j_{2}}\right)}_{1\leq j_{1},j_{2}\leq J}.$$

The PCBS method can be implemented using the approximate covariance matrices as in (10) and (11).

### Imputation based methods in the presence of missing summary statistics

When the contributed summary association statistics from participating studies contain missing values, a natural strategy is to replace the missing values using imputation. Several imputation methods were previously developed. One method is REPLACE0, which is to replace the missing values by 0. We denote the resulting statistics as **U**^**0**^ and **V**^**0**^. To mathematically describe this method, we define an indicator variable *M*_*jk*_, which takes value 1 if the summary statistics at site *j* in study *k* is measured and 0 if missing. The meta-analysis score statistic is calculated by

$$U_{j}^{0}=\sum_{k\in \left\{k:M_{jk}=1\right\}}U_{jk}\;\mathrm{and}\;V_{j_{1}j_{2}}^{0}=\sum_{k\in \left\{k:M_{j_{1}k}=M_{j_{2}k}=1\right\}}V_{j_{1}j_{2}k}$$

We proved in **S1 Text** that replacing missing summary association statistics with zero will bias the genetic effect estimate, i.e. $E(U_{G^{*}}^{0})\neq V_{G^{*}}^{0}\;\beta_{G^{*}}$. As a consequence, under the null hypothesis that the candidate variant is not associated with the phenotype, the expectation of the conditional score statistics is not equal to 0, i.e. $E(U_{G|G^{*}})=V_{GG^{*}}\beta_{G^{*}}-V_{GG^{*}}^{0}{\left(V_{G^{*}}^{0}\right)}^{-1}E(U_{G^{*}}^{0})\neq 0$. The type I error for conditional analysis can be highly inflated.

A more sophisticated set of methods is to impute missing summary statistics based upon LD information. Yet, the genetic effect estimates based upon the imputed Z-score statistics are often biased, unless the following condition holds

$$E[Z_{imp}]=\Sigma_{imp,tag}\Sigma_{tag}^{-1}{E[Z}_{tag}]$$

where *Z*_*imp*_ and *Z*_*tag*_ are Z-score statistics at the missing and tagSNP sites, Σ_*imp*,*tag*_ and Σ_*tag*_ are genotype correlation matrices. A special case for this condition is that both the tagSNP and missing variants have null effects. Similar to REPLACE0, applying impG+meta method can lead to inflated type I errors.

#### DISCARD method

An alternative approach we call DISCARD, is to remove studies with missing summary statistics and only use studies with complete data. The meta-analysis score statistics under this analysis strategy are given by:

$$U_{j}^{rm}=\sum_{k\in \{{k:M}_{jk}=1,\forall j\}}U_{jk},\;V_{j_{1}j_{2}}^{rm}=\sum_{k\in \{{k:M}_{jk}=1,\forall j\}}V_{j_{1}j_{2}k}$$

An obvious limitation of the DISCARD method is that it may result in the removal of a large number of studies and a significant loss of power.

#### SYN+ method–Extension of synthesis method to meta-analysis of genetic association studies

Synthesis methods have been developed in the statistics literature for combining the joint effects of multiple predictors in a meta-analysis [13]. The method can handle the scenario where different studies measure different sets of predictors. The published methods only considered the simplest scenarios where at least one study measures the full set of variables. Additionally, the published synthesis methods lack an implementation that can be applied in genetic association meta-analysis.

Our extension, the SYN+ method includes the following steps:

1. Derive estimating equations using marginal SNP effects
  The joint effect needed by the synthesis method can be obtained using the shared score statistics and their covariance matrices. To facilitate the presentation of the method, we re-write the full model, separating the measured ($G_{\left\{j:M_{jk}=1\right\}}$) and unmeasured variants ($G_{\left\{j:M_{jk}=0\right\}}$). The full model is given by
  $$Y_{k}=G_{\left\{j:M_{jk}=1\right\}}\beta_{\left\{j:M_{jk}=1\right\}}+G_{\left\{j:M_{jk}=0\right\}}\beta_{\left\{j:M_{jk}=0\right\}}+Z_{k}\gamma_{k}+\epsilon$$ (Mfull)
  The residual error from M_full_ satisfies $\epsilon ∼N(0,\sigma_{f}^{2})$.
  The score statistics from the measured variants satisfy
  $$E\left(V_{\{j_{1},j_{2}:M_{j_{1}k}=M_{j_{2}k}=1\},k}^{-1}U_{\{j:M_{jk}=1\},k}\right)=\beta_{\left\{j:M_{jk}=1\right\}}+V_{\{j_{1},j_{2}:M_{j_{1}k}=M_{j_{2}k}=1\},k}^{-1}V_{\{j_{1},j_{2}:M_{j_{1}k}=1,M_{j_{2}k}=0\},k}\beta_{\left\{j:M_{jk}=0\right\}}$$ (12)
  The formula (12) can be viewed as an estimating equation for the unknown parameters **β**.
2. Estimate covariance matrix between genetic effects:
  To be able to fit the estimating Eq in (12), we need to estimate the covariance matrix between score statistics, from both the measured variants and the unmeasured variants. For exact conditional analysis, the covariance matrix can be estimated using formula (8) and (9). For approximate conditional analysis, the same method can be used with the approximate covariance matrix ${\tilde{V}}_{k}$
3. Estimate the joint effects by regression:
  As in the original synthesis method, the joint effects **β** can be estimated by the estimating Eq in (12).
  The estimating Eq (12) uses the joint effects of the measured variants from each participating study as input. The variance of $V_{\{j_{1},j_{2}:M_{j_{1}k}=M_{j_{2}k}=1\},k}^{-1}U_{\{j:M_{jk}=1\},k}$ is influenced by both the residual variance from model M_full_ as well as the phenotypic variance explained by the unmeasured variants in study *k*. When missing rate is higher or when the unmeasured variants have larger effects, $V_{\{j_{1},j_{2}:M_{j_{1}k}=M_{j_{2}k}=1\},k}^{-1}U_{\{j:M_{jk}=1\},k}$ can be noisier and have larger variance. PCBS, on the other hand, aggregates information from all studies, and jointly models the effects of all variants (including the variants that may be missing from a particular study but measured in others). Therefore, PCBS is statistically more efficient than SYN+, even though the SYN+ method gives unbiased estimates of the joint effect. The power for the SYN+ method can be much lower than PCBS when the missing rate is high or when the conditioned variants explain a larger fraction of the trait variance.

### Simulation study

We conducted extensive simulations to evaluate the performance of PCBS as well as 5 alternative approaches, including 1) impG+meta; 2) COJO; 3) REPLACE0; 4) DISCARD and 5) SYN+ using simulated data. We simulated genetic data following a coalescent model that we previously used for evaluating rare variant association analysis methods [2]. The model captures an ancient population bottleneck and recent explosive population growth. Model parameters were tuned such that the site frequency spectrum and the fraction of the singletons of the simulated data match that of large scale sequence datasets.

For quantitative traits, phenotype data from each cohort were simulated according to the linear model:

$$Y_{i}=\beta_{0}+\sum_{j=1}^{J}G_{ij}\beta_{j}+\sum_{j=1}^{J}G_{ij}^{*}\gamma_{j}+\epsilon_{i}$$

where *G*_*ij*_ and $G_{ij}^{*}$ denote the candidate and conditioned variant genotypes, and *β*_*j*_ and *γ*_*j*_ are their effects respectively. The model assumes that the genetic variants have additive effects on the phenotype.

The genetic effects for candidate variants follow a mixture normal distribution, which accommodates the possibility that a genetic variant can be causal (with probability *c*) or non-causal (with probability 1 − *c*): $\beta_{j}∼(1-c)\times I(0)+c\times N(0,\tau_{\beta }^{2})$. The genetic effects for the conditioned variants follow: $\gamma_{j}∼N(0,\tau_{\gamma }^{2})$.

To evaluate the influence of missing data, we randomly chose a certain fraction (10% 30% or 50%) of the sites from each study and masked them as missing. We then applied the new method PCBS, along with impG+meta, COJO, DISCARD, REPLACE0 and SYN+ to the data. In our evaluations, we used the exact LD with COJO and impG+meta, in order to remove the influence of approximate LD and focus on the impact of missing summary statistics on the power and type I error. We evaluated the type I errors and power for each approach under a variety of scenarios with different genetic effect sizes, fractions of causal variants in the gene region, and the fractions of missing data.

### Meta-analysis of datasets with cigarettes per day phenotype

To evaluate the effectiveness of methods in real datasets, we applied our methods to a meta-analysis of seven cohorts with a cigarettes-per-day (CPD) phenotype, a key measurement for studying nicotine dependence. Participating studies were the Minnesota Center for Twin and Family Research (MCTFR) [17–19], SardiNIA[20], METabolic Syndrome In Men (METSIM)[21], Genes for Good [22], COPDGene with samples of European ancestry[23], Center for Antisocial Drug Dependence (CADD) [24], and full UK Biobank. Genotypes were imputed using the Haplotype Reference Consortium panel [25] and the Michigan Imputation Server [26] (with the exception of UK Biobank dataset, which was imputed centrally by the UK Biobank team). Summary association statistics from the seven cohorts were generated using RVTESTS [27], and meta-analysis performed using rareMETALS with the PCBS statistics and other alternative approaches. Detailed descriptions of the cohorts are available in **S1 Text** section 4, including the methods for association analyses and the adjusted covariates.

To ensure the validity of our association analysis results, we conducted extensive quality control for the imputed genotype data. We filtered out variant sites with the imputation quality metric *R*^2^ < .7, and sites that showed large differences in allele frequencies from the imputation reference panel. Imputation dosages were used in the association analysis.

For each sentinel SNP with genome-wide significance (α = 5×10^−8^), we defined the locus as the 1 MB window surrounding it. We applied iterative single variant conditional analysis to identify independently associated variants in each locus. We started by conditioning on the most significant variant from marginal association analysis. After each round of the association analysis, if the top variant remained statistically significant, we added the top variant to the set of conditioned variants, and performed an additional round of association testing. We applied the six methods to analyze the data, including the PCBS statistic, SYN+, impG+meta, REPLACE0, DISCARD and COJO. In order to examine if the low frequency variants in aggregate can be explained by the identified independently associated variants, we also performed gene-level association analysis for rare variants with MAF<1%, conditional on the identified independently associated variants.

## Results

### Evaluation of type I error

We evaluated the type I errors for the six conditional analysis methods PCBS, SYN+, COJO (with exact LD), impG+meta, REPLACE0, and DISCARD. Scenarios were considered for different combinations of the fractions of missing data, the genetic effects of the variants in the candidate gene, and the genetic effects of the conditioned variants.

First, we noted that PCBS, SYN+ and DISCARD are the only three methods that have controlled type I errors across all scenarios, consistent with our theoretical expectation (**Table 1**). The type I error rate for the other three methods, i.e. impG+meta, REPLACE0 and COJO are inflated in a number of scenarios. The inflation tends to increase with the effect of the conditioned variant(s) and the rate of missingness. In many scenarios, the type I error can be >100X inflated over the significance threshold (α = 5×10^−8^). For example, when the conditioned variant effect is .04, and the association statistics from 30% of the variant sites are missing, type I errors for impG+meta, COJO and REPLACE0 are .015, .57 and .74 under the significance threshold of *α* = 0.005. When the missing rate is 50%, and the conditioned variant effects is .08, the type I errors for the three methods become .25, .65, and .60.

**Table 1:** Power and type I errors of meta-analysis of single variant tests in the presence of missing data for continuous outcomes. Datasets were simulated according to the genetic and phenotype model described in METHODS. Meta-analysis was performed to combine 20 cohorts with 1500 individuals each. For each replicate, summary association statistics were generated, and a certain fraction of the generated summary statistics were masked as missing. Scenarios with different combinations of known variant effects, candidate variant effects and fractions of missingness were considered. Six analysis strategies were considered: 1) PCBS; 2) SYN+; 3) ImpG+meta; 4) COJO; 5) DISCARD and 6) REPLACE0. Type I error and power were evaluated using 10^5^ replicates under the significance threshold of *α* = 0.005.

Conditioned Variant Effect	Candidate Variant Effect	Fraction of Missing Data	Type I Error/Power						
			PCBS	SYN+	ImpG+Meta	COJO	DISCARD	REPLACE0	Analyze the Full Dataset [Gold Standard]
			Type I Error						
0.04	0	0.1	5.0 × 10^−3^	4.4 × 10^−3^	5.2 × 10^−3^	0.065	4.1 × 10^−3^	9.5 × 10^−3^	4.9 × 10^−3^
0.04	0	0.3	5.4 × 10^−3^	4.0 × 10^−3^	0.015	0.57	3.8 × 10^−3^	0.14	5.4 × 10^−3^
0.04	0	0.5	5.2 × 10^−3^	3.5 × 10^−3^	0.021	0.61	1.8 × 10^−3^	0.46	5.1 × 10^−3^
0.08	0	0.1	5.0 × 10^−3^	3.0 × 10^−3^	9.3 × 10^−3^	0.25	2.0 × 10^−3^	0.025	4.8 × 10^−3^
0.08	0	0.3	5.6 × 10^−3^	1.7 × 10^−3^	0.12	0.61	2.0 × 10^−3^	0.45	4.4 × 10^−3^
0.08	0	0.5	5.2 × 10^−3^	1.3 × 10^−3^	0.25	0.65	9.3 × 10^−4^	0.60	4.9 × 10^−3^
			Power						
0.04	0.04	0.1	0.22	0.20	-	-	0.092	-	0.22
0.04	0.04	0.3	0.21	0.18	-	-	0.021	-	
0.04	0.04	0.5	0.20	0.17	-	-	4.5 × 10^−3^	-	
0.08	0.04	0.1	0.21	0.17	-	-	0.063	-	0.21
0.08	0.04	0.3	0.21	0.12	-	-	0.013	-	
0.08	0.04	0.5	0.19	0.11	-	-	3.2 × 10^−3^	-	
0.04	0.08	0.1	0.88	0.87	-	-	0.57	-	0.88
0.04	0.08	0.3	0.87	0.85	-	-	0.12	-	
0.04	0.08	0.5	0.86	0.83	-	-	0.017	-	
0.08	0.08	0.1	0.88	0.84	-	-	0.49	-	0.88
0.08	0.08	0.3	0.86	0.76	-	-	0.083	-	
0.08	0.08	0.5	0.83	0.74	-	-	0.011	-	

Second, among the methods with the controlled type I error rates (i.e. SYN+, PCBS and DISCARD), PCBS is consistently the most powerful method (**Table 1**). The power advantage of PCBS over the other two approaches increases when 1) the conditioned variant(s) have larger effects or 2) the fraction of missing summary association statistics is larger. For example, when candidate variant effect is .04, the conditioned variant effect is .08, and the missing rate of score statistics is 30%, the power for PCBS is .21, which is 75% higher than the power for SYN+ (.12). When the candidate variant effect is.08, the conditioned variant effect is .08, and score statistics from 50% of the variant sites in each participating study are missing, the power for PCBS and SYN+ are respectively .83 and .74.

Due to the obvious limitations of complete case analysis, the DISCARD method of discarding the studies with missing data can lead to considerable loss of power (**Table 1**). The power for DISCARD is substantially lower than PCBS and SYN+. In some scenarios where the missingness is high, the power is barely larger than the significance threshold.

Interestingly, gene-level association tests are affected by two types of missing data with opposite consequences: Missing values at causal variant sites reduce power but missing values at non-causal variant sites tend to reduce noise and thus improve power (**Table 2**). When missingness is higher, the power of gene-level tests is lower, but the power loss is small. For instance, when a causal variant in the candidate gene has effects sampled from *N*(0,0.2^2^), the conditioned variant has effect .1, and 30% of the contributed summary statistics in each study have missing values, the power for burden/SKAT/VT tests are 58%/58%/56%, which are only slightly reduced compared to the power of analyzing the complete datasets (60%/61%/60%). On the other hand, the method that discards studies with missing data has much reduced power (0.011/0.011/8.8×10^−3^).

**Table 2:** Power and type I errors of meta-analysis of gene-level tests in the presence of missing data. Datasets were simulated according to the genetic and phenotype model described in METHODS. Within the gene region, 20% of the variant sites are deemed causal. Meta-analysis was performed to combine 10 cohorts with 2000 individuals each. For each replicate, summary association statistics were generated, and a certain fraction (10%, 30% or 50%) of the generated summary statistics were masked as missing. Scenarios with different combinations of known variant effect, candidate variant effects and fractions of missingness were considered. To evaluate the power loss due to missing data, we also analyzed the full dataset as a gold standard. Type I errors and power were evaluated for three rare variant tests (simple burden, SKAT and VT) using 1 million replicates under the significance threshold of *α* = 0.005.

Conditioned Variant Effect	Candidate Variant Effect (*τ*_*β*_)	Fraction of Missing Data	Type I Error/Power for Burden/SKAT/VT (α = 0.0005)	
			PCBS	Analyze the Full Dataset [Gold Standard]
**Type I Error**				
0.05	0	0.1	4.5 × 10^−3^/3.1 × 10^−3^/3.8 × 10^−3^	4.8 × 10^−3^/4.1 × 10^−3^/4.5 × 10^−3^
0.05	0	0.3	4.7 × 10^−3^/4.4 × 10^−3^/3.4 × 10^−3^	4.7 × 10^−3^/4.4 × 10^−3^/6.0 × 10^−3^
0.05	0	0.5	6.4 × 10^−3^/4.0 × 10^−3^/3.4 × 10^−3^	4.7 × 10^−3^/5.0 × 10^−3^/4.4 × 10^−3^
0.1	0	0.1	3.3 × 10^−3^/2.6 × 10^−3^/4.9 × 10^−3^	5.3 × 10^−3^/5.9 × 10^−3^/5.3 × 10^−3^
0.1	0	0.3	6.0 × 10^−3^/4.7 × 10^−3^/4.1 × 10^−3^	4.7 × 10^−3^/5.4 × 10^−3^/4.1 × 10^−3^
0.1	0	0.5	6.3 × 10^−3^/6.7 × 10^−3^/6.3 × 10^−3^	5.8 × 10^−3^/5.9 × 10^−3^/4.9 × 10^−3^
**Power**				
0.05	0.1	0.1	0.21/0.21/0.19	0.22/0.23/0.21
0.05	0.1	0.3	0.19/0.19/0.17	
0.05	0.1	0.5	0.17/0.16/0.14	
0.1	0.1	0.1	0.22/0.22/0.20	
0.1	0.1	0.3	0.20/0.20/0.18	
0.1	0.1	0.5	0.17/0.16/0.14	
0.05	0.2	0.1	0.59/0.60/0.58	0.60/0.61/0.59
0.05	0.2	0.3	0.57/0.57/0.55	
0.05	0.2	0.5	0.54/0.53/0.52	
0.1	0.2	0.1	0.59/0.60/0.58	
0.1	0.2	0.3	0.58/0.58/0.56	
0.1	0.2	0.5	0.54/0.53/0.52	

Our method was developed for the fixed effect meta-analysis, where the genetic effects are assumed to be constant across different studies. But since PCBS first aggregates association statistics from across studies and then performs conditional analysis, the impact of genetic effects heterogeneities does not invalidate the test and the type I error remains well controlled. The power is slightly reduced, but the advantages over other methods remain. To confirm this, we performed simulation analysis assuming that the genetic effects across studies are heterogeneous (**S1 Table, S2 Table**). In our simulations, the genetic effects for a given variant in different studies were simulated from a normal distribution $N\left(\mu_{\beta_{G^{*}}},{\left(\mu_{\beta_{G^{*}}}/2\right)}^{2}\right)$, allowing for substantial between-study heterogeneities. The power comparison for different methods remains similar to the scenarios where the genetic effects are the same across studies.

### Results for the meta-analysis of cigarettes per day phenotype

We performed a meta-analysis of CPD phenotype in 7 cohorts. The locus *CHRNA5*-*CHRNB4*-*CHRNA3* was previously identified as associated with CPD [28]. After careful quality control, 42,669,770 variants were meta-analyzed. A majority (32,796,258) of these variants had minor allele frequencies <1%.

It is important to note that even with high quality imputation panels, such as the haplotype reference consortium panel [25], there was still considerable missing data in the imputed datasets. A fraction of 76.1% of the variants were missing from at least one participating study post imputation, due to filtering on the imputation quality (R^2^>.7). Compared to common variants, rare variants were considerably more likely to be missing: 95.3% of the variants with MAF<1% were missing from at least one cohort, compared to the fraction of 20.1% for the common variants with MAF>1%.

The Quantile-Quantile plot for–log_10_(p-value) is well calibrated (**S1 Fig**). The genomic control value is 1.14 for common variants with MAF>0.01, and 1.00 for rare variants with MAF<0.01. The genomic control value is consistent with that of large scale GWAS for highly polygenic traits [29, 30]. The intercept for LD score regression [31] was 1.01, which shows little influence from potential population structure. The meta-analysis of 7 cohorts identifies 9 loci (**S2 Fig**), including the well-known CPD associated loci, the nicotine receptor genes *CHRNB2*, *CHRNB3*-*CHRNA6*, *CHRNA5*-*CHRNB4*-*CHRNA3*, the gene *CYP2A6* that encodes cytochrome P450 protein, the gene *PDE1C* that encodes Phosphodiesterase 1C, *FAM163B-DBH*, *YTHDF3* and *GRM4*. Among these loci, *CHRNB2* and *FAM163B-DBH* are associated with CPD at the genome-wide significance threshold for the first time.

While smoking behaviors are known to be heritable, only the *CHRNA5*-*CHRNB4*-*CHRNA3* and *CYP2A6* loci have been consistently implicated in human GWAS to date. The other nicotine receptor gene *CHRNB3-CHRNA6* was first identified with genome-wide significance in an isolated population for associations with nicotine dependence and nicotine use [32]. *CHRNB2* was implicated in the nicotine dependence trait, but not at genome-wide significance. To our knowledge, there is no report that this gene is associated with CPD at genome-wide significance [33].

In order to understand the allelic architecture of the CPD phenotype and compare different methods on real data, we performed sequential forward selection with the new PCBS method, and identified 5 independently associated variants for the *CHRNA5*-*CHRNB4*-*CHRNA3* locus and 4 independently associated variants for the CYP2A6 locus at genome-wide significance threshold (with p-values < 5 × 10^−8^) (**Table 3**). The other loci do not have additional independently associated variants besides the sentinel variant.

**Table 3:** Independently associated variants identified using sequential forward selection with PCBS method. Sequential conditional analyses for the 9 loci were conducted, where we iteratively performed conditional analysis, conditioning on the top variants from earlier rounds. Top association signals at each iteration are shown. The sequential conditional analysis stops when the top association signal is no longer significant under the genome-wide significance threshold *α* = 5 × 10^−8^.

POS	RS	REF	ALT	AF	PVALUE	BETA	SE	N		ANNO	GENE
Locus rs2072659 Marginal association analysis											
**1:154548521**	rs2072659	C	G	0.1	1.9 × 10^−8^	-0.041	7.3 × 10^−3^	134862		Utr3	*CHRNB2*
Locus rs550432263 Marginal association analysis											
**5:1385253**	rs550432263	G	A	2.8 × 10^−6^	3.6 × 10^−8^	71	13	34858		Intergenic	*SLC6A3*
Locus rs9366836 Marginal association analysis											
**6:34009601**	rs9366836	A	G	0.17	3.3 × 10^−8^	0.028	5.2 × 10^−3^	134862		Intron	*GRM4*
Locus rs215600 Marginal association analysis											
**7:32333642**	rs215600	G	A	0.64	4.8 × 10^−11^	-0.027	4.0 × 10^−3^	134862		Intron	*PDE1C*
Locus rs58379124 Marginal association analysis											
**8:42579203**	rs58379124	T	C	0.77	4.4 × 10^−14^	0.035	4.6 × 10^−3^	134862		Intron	*CHRNB3*
Locus rs1217106 Marginal association analysis											
**8:64567670**	rs1217106	A	G	0.78	2.2 × 10^−9^	-0.028	4.4 × 10^−3^	134862		Intergenic	*YTHDF3*
Locus rs56116178 Marginal association analysis											
**9:136460224**	rs56116178	A	G	0.11	2.5 × 10^−9^	0.038	6.3 × 10^−3^	134862		Intergenic	*FAM163B-DBH*
Locus rs11852372 Marginal association analysis											
**15:78801394**	rs11852372	A	C	0.34	7.7 × 10^−115^	0.096	4.2 × 10^−3^	128249		Intron	*AGPHD1*
Conditional on rs11852372											
**15:78896129**	rs1317286	A	G	0.34	1.7 × 10^−22^	0.027	2.8 × 10^−3^	128249		Intron	*CHRNA3*
Conditional on rs11852372 and rs1317286											
**15:78814389**	rs7181245	C	T	0.21	2.5 × 10^−13^	-0.032	4.4 × 10^−3^	128249		Intron	*AGPHD1*
Conditional on rs11852372, rs1317286 and rs7181245											
**15:78911181**	rs8040868	T	C	0.40	2.2 × 10^−11^	0.020	2.9 × 10^−3^	128249	Synonymous		*CHRNA3*
Conditional on rs11852372, rs1317286, rs7181245 and rs8040868											
**15:78739763**	rs2089162	A	G	0.33	3.5 × 10^−8^	0.011	2.0 × 10^−3^	128249		Intron	*IREB2*
Locus rs56113850 Marginal association analysis											
**19:41353107**	rs56113850	T	C	0.58	6.6 × 10^−67^	0.070	4.0 × 10^−3^	128249		Intron	*CYP2A6*
Conditional on rs56113850											
**19:41371480**	rs117824460	A	G	0.029	6.2 × 10^−23^	-0.13	0.013	128249		Intergenic	*CYP2A6*
Conditional on rs56113850 and rs117824460											
**19:41406448**	rs117540499	G	A	0.023	2.4 × 10^−17^	-0.11	0.013	128249		Intergenic	*CYP2A6*
Conditional on rs56113850, rs117824460 and rs117540499											
**19:41345395**	rs7246742	T	G	0.13	1.9 × 10^−8^	-0.033	5.9 × 10^−3^	128249		Intergenic	*CYP2A6*

As a comparison, we also performed sequential forward selection using the five alternative approaches (**S3 Table**). Using the SYN+ method, fewer independently associated variants are identified. At the *CHRNA5*-*CHRNB4*-*CHRNA3* locus, 3 independently associated variants are identified, and also at the *CYP2A6* locus, only 3 independently associated variants are identified. DISCARD also identifies fewer number of independently associated SNPs. The results from real data analysis is consistent with our simulation study that PCBS has higher power than alternative approaches.

Among the approaches that have inflated type I errors in simulations, impG+meta identifies a lot of SNPs with very significant p-values. Many of these identified SNPs have substantial missingness among the participating cohorts (e.g. N<50,000). Given the inflated type I errors that we observed in simulations, as well as the small available sample sizes for the top variants, the validity of the results using impG+meta is of concern. Most of the top variants identified by COJO and REPLACE0 have low missingness, so there are not many false positive results. Yet, COJO and REPLACE0 identified fewer independently associated SNPs compared to PCBS and SYN+ (**Table 3** and **S3 Table**). Together, the analysis of real data confirmed our simulation experiments.

We examined if our independently associated variants explained previously known association signals. To do this, we looked up GWAS catalog [34] using key words “CPD” or “cigarettes per day” and found 11 associated variants in the loci that we identified (**S4 Table**). We first analyzed these 11 variants conditional on our independently associated variants. All of these variants became insignificant, which indicated that our newly identified independently associated variants can explain previously known association signals. We also performed conditional analysis in the opposite direction to examine if our identified association signal may be explained by the known variants. We found that variants within the *CPY2A6* locus remained highly significant and variants within the *CHRNA5-CHRNB4-CHRNA3* locus remained marginally significant. Together, our independently associated variants explained 1.1% of the phenotypic variance, which substantially improves the phenotypic variance (.64%) explained by the 11 known signals.

Finally, in addition to single variant association, we investigated if rare variants within each of the 9 loci were independently associated with the CPD phenotype (**S5 Table**). 27 genes were analyzed using simple burden, SKAT and VT tests under a MAF threshold of 0.01. Only one gene (*CHRNA5*) has gene-level p-values less than 0.05/27, which is the Bonferroni threshold. None of the genes have exome-wide significant gene-level association p-values.

## Discussion

We proposed a simple yet effective meta-analysis method to estimate joint and conditional effects of rare variants in the presence of missing summary statistics from contributing studies. The method leads to the optimal use of shared summary association statistics. It has well controlled type I error and much higher power than alternative approaches even when a large number of contributing studies contain missing summary statistics.

Several approaches were previously developed to combine genetic effects across studies when different studies may measure different genetic variants e.g. Verzilli et al [35] and Newcombe et al [36]. These methods have some noticeable limitations. The method by Verzilli et al requires the individual level genotype and phenotype data as input. Also the method focuses on random effects meta-analysis, while our approach focuses on fixed effect meta-analysis. The method by Newcombe et al models the haplotype counts in cases and controls. The method does not allow for the adjustment of covariates, which is a serious limitation. Both methods use MCMC for fitting the model, which may not scale well for contemporary meta-analysis with tens of millions of variants and dozens of studies.

It is important to note that our method, PCBS is developed for proper conditional and joint analysis when imputation fails to work. As we showed in our meta-analysis of smoking phenotypes, even with the state-of-the-art imputation methods and high quality reference panels, there are still considerable amount of association statistics filtered out from participating studies. The rate of missingness is much higher for rare variant association statistics than for common variant association statistics. PCBS will be particularly useful for the meta-analysis of sequence data, where the measured variants are predominantly low frequency or rare [37].

Our method is not developed to replace genotype imputation. Genotype imputation fills in missing genotypes with imputed values, and increases effective sample sizes and power. Our method does not increase the effective sample size for tested variants. In practice, imputation method should first be applied in each participating cohort. Our method should be applied at the meta-analysis stage for valid and powerful conditional meta-analysis, especially when contributed summary statistics from participating cohorts contain missing values.

Missing data will continue to be a persistent issue in the next generation of large-scale genetic studies. Major biobanks have started to develop their own genotyping arrays and imputation reference panels to incorporate customized content. Combining these newly genotyped studies with existing datasets will result in missing summary statistics. Our method will continue to be useful when analyzing these newly generated datasets.

Another major application of the proposed method is in the meta-analysis of sequence data. Given the use of targeted sequencing assays and variability in batch processing and quality control across studies, it would be difficult to impute missing genotype data or missing summary statistics. One of the challenges in sequence-based meta-analysis is to properly represent monomorphic sites, as the polymorphic variant sites are not known a priori. Neither un-called variant sites (e.g. due to insufficient coverage or failed quality control) nor monomorphic sites contribute to the single variant meta-analysis statistic. Yet they should be treated differently in joint and conditional meta-analysis. Summary statistics from monomorphic variants should be replaced by zeros. On the other hand, summary statistics from un-called variants should be treated as missing data, and the conditional association analysis can be performed using our partial correlation based score statistics.

While not the focus of this article, the proposed method is also helpful for downstream analyses that make use of the joint effects of multiple variants, e.g. estimating the phenotypic variance explained by variants in LD or fine mapping causal variants (e.g. using methods such as RIVERA [38], FINEMAP [39], CAVIARBF [40]) The validity of these analyses relies critically on the proper estimates of the joint effects, which are usually obtained from single variant association statistics and the LD information from a reference panel. When summary statistics from contributing studies contain missing data, the correlations between resulting marginal meta-analysis association statistics may not be properly approximated by the LD estimated from a reference panel. In this case, PCBS can be used to obtain valid joint effect estimates, which can potentially lead to better calibrated estimates phenotypic variance explained and more accurate fine mapping analysis.

Taken together, our partial correlation based score statistic is a simple yet effective method for estimating joint and conditional effects from a meta-analysis. With its efficient implementations in RVTESTS, RAREMETAL and rareGWAMA, this method will have broad application in current array-based meta-analysis, as well as the upcoming imputation-based meta-analysis (e.g. based upon the haplotype reference consortium panel) and sequence-based meta-analysis. Correct inference on the joint and conditional effects using these methods will pave the way for a more accurate characterization and a more complete understanding of the genetic architecture of complex traits.

## Supporting information

S1 Text (DOCX)

S1 Fig Quantile-quantile plot of–log_10_(p-value) for the meta-analysis of cigarettes per day phenotype. Genomic control values were separately reported for variants with MAF>0.01 and with MAF<0.01.
(TIFF)

S2 Fig Manhattan plot for the meta-analysis of cigarettes per day phenotype. (TIFF)

S1 TablePower and type I errors of meta-analysis of single variant tests in the presence of missing data and genetic effect heterogeneity.We evaluated the impact of large heterogeneity in the genetic effects on the power and type I errors for the PCBS statistics. The effects of the conditioned variants in each cohort are sampled from the distribution $N(\mu_{\beta_{2}},{\left(\mu_{\beta_{2}}/2\right)}^{2})$. All other simulation settings are the same as in Table 1.(DOCX)

S2 TablePower and type I errors of meta-analysis of gene-level tests in the presence of missing data and genetic effect heterogeneity.We evaluated the impact of large heterogeneity in the genetic effects on the power and type I errors for the PCBS statistics. The genetic effects for the conditioned variants in each cohort are sampled from the distribution $N\left(\mu_{\beta_{2}},{\left(\mu_{\beta_{2}}/2\right)}^{2}\right)$. All other simulation settings are the same as in Table 2.(DOCX)

S3 Table Results of sequential forward selection using the alternative methods including SYN+, COJO, impG+meta, REPLACE0, DISCARD. (XLSX)

S4 Table Two way conditional analysis of independently associated variants and previously reported GWAS hits at *CHRNA5*-*CHRNB4*-*CHRNA3* locus and the *CYP2A6* locus. We analyzed independently associated variants from the meta-analysis conditional on the previously known GWAS signals (Panel A). We also performed the conditional analyses in the opposite direction, where we analyzed the previously reported GWAS variants conditional on the identified independently associated variants (Panel B).
(XLSX)

S5 Table Gene-level conditional analysis results. We analyzed gene-level association conditional on the independently associated variants in the same loci, which were identified using sequential forward selection. Three gene level association tests were performed, including simple burden tests, SKAT and VT. No significant gene-level associations were identified (with p<0.05/27).
(XLSX)
