# Supplementary material for: Proper conditional analysis in the presence of missing data: Application to large scale meta-analysis of tobacco use phenotypes
Source: PLoS Genet. 2018 Jul 17;14(7):e1007452. doi: 10.1371/journal.pgen.1007452 (PMC6063450; doi:10.1371/journal.pgen.1007452)
Supplement: S1 Text — (DOCX) [file pgen.1007452.s001.docx]

**S1 Text**

**Proper Conditional Analysis in the Presence of Missing Data: Application to Large Scale Meta-analysis of Tobacco Use Phenotypes**

**1. Replace Missing Summary Statistics with 0 Will Lead to Biased Genetic Effect Estimates**

In the presence of missing data, one possible strategy is to replace missing summary statistics with zero. We denote the resulting score statistics and their covariance matrices as $\mathbf{U}^{\mathbf{0}}$ and $\mathbf{V}^{\mathbf{0}}$. Below, we will demonstrate theoretically that replacing missing summary statistics with zero will lead to biased genetic effect estimates.

When replacing missing summary statistics with 0, it is easy to verify that the score statistics $\mathbf{U}_{\mathbf{k}}^{\mathbf{0}}$ from study $k$ still[1] follow the normal distribution:

$\mathbf{U}_{\mathbf{k}}^{\mathbf{0}}\sim\mathrm{MVN}\left( \boldsymbol{0},\mathbf{V}_{\mathbf{k}}^{\mathbf{0}} \right)$ (S1)

The meta-analysis score statistics satisfy $\mathbf{U}^{\mathbf{0}}\sim\mathrm{MVN}\left( \boldsymbol{0},\mathbf{V}^{\mathbf{0}} \right)$. As a result, the marginal association analysis using $\mathbf{U}^{\mathbf{0}}$ and $\mathbf{V}^{\mathbf{0}}$ will be valid.

We want to prove that under the alternative hypothesis $E\left( \mathbf{U}_{\mathbf{k}}^{\mathbf{0}} \right)\neq\mathbf{V}_{\mathbf{k}}^{\mathbf{0}}\boldsymbol{\beta}$**.** We prove this by contradiction. To facilitate the presentation, we denote the underlying score statistic in study $k$ as $U_{jk}^{*}, V_{jj^{'}k}^{*}$, $1\leq j,j^{'}\leq J$. Using this notation, we can represent the contributed score statistic as $U_{jk}^{0}=U_{jk}^{*}M_{jk}$, $V_{jj^{'}k}^{0}=V_{jj^{'}k}^{*}M_{jk}M_{j^{'}k}$. Without loss of generality, we assume that the association statistic at the 1^st^ variant site is measured, and the score statistics from at least one variant site are missing. Given that the score statistic at site 1 is measured, $U_{1k}^{0}=U_{1k}^{*}$. If $E\left( U_{1k}^{0} \right)=\sum_{j^{'}} V_{1j^{'}k}^{0}\beta_{j^{'}}$ holds for all possible $\beta_{j^{'}}$’s, it follows that $\sum_{j^{'}} V_{1j^{'}k}^{*}M_{j^{'}k}\beta_{j^{'}}=\sum_{j^{'}} V_{1j^{'}k}^{*}\beta_{j^{'}}$ for arbitrary choices of $\beta_{j^{'}}$. So all $M_{j^{'}k}$’s must be 1, which contracts the assumption that summary statistics from at least one variant site is missing. Therefore, the genetic effect calculated from meta-analysis statistics, i.e. ${\hat{\boldsymbol{\beta}}}^{\mathbf{0}}=\left( \mathbf{V}^{\mathbf{0}} \right)^{-1}\mathbf{U}^{\mathbf{0}}$ will be biased, which will lead to inflated type I errors in conditional analysis.

**2. Consistent Estimator for Joint Effects in the Presence of Missing Data**

In this section, we will outline the derivation of the consistent estimator for the joint effects of candidate and conditioned variants in the presence of missing data. In study $k$, the following model is used to analyze the genetic association

$\mathbf{Y}_{\mathbf{k}}=\mathbf{G}_{\mathbf{jk}}\boldsymbol{\beta}_{\mathbf{k}}+\mathbf{Z}_{\mathbf{k}}\boldsymbol{\gamma}_{\mathbf{k}}+\boldsymbol{\epsilon}_{\mathbf{k}}$ (S2)

The score statistics and their variance-covariances from the $k^{th}$ study are equal to

$$U_{jk}=\frac{1}{\hat{\sigma}_{k}^{2}}\mathbf{G}_{\mathbf{jk}}^{\mathbf{T}}\left( I-\left( \mathbf{Z}_{\mathbf{k}}^{\mathbf{T}}\mathbf{Z}_{\mathbf{k}} \right)^{-1}\mathbf{Z}_{\mathbf{k}}^{\mathbf{T}} \right)\mathbf{Y}_{\mathbf{k}}$$

$$V_{j_{1}j_{2}k}=\frac{1}{\hat{\sigma}_{k}^{2}}\mathbf{G}_{\mathbf{j}_{\mathbf{1}}\mathbf{k}}^{\mathbf{T}}\left[ \mathbf{I}-\mathbf{Z}_{\mathbf{k}}\left( \mathbf{Z}_{\mathbf{k}}^{\mathbf{T}}\mathbf{Z}_{\mathbf{k}} \right)^{-1}\mathbf{Z}_{\mathbf{k}}^{\mathbf{T}} \right]\mathbf{G}_{\mathbf{j}_{\mathbf{2}}\mathbf{k}}$$

Under model (S2), the phenotype follows the normal distribution: $\mathbf{Y}_{\mathbf{k}}\sim\mathrm{MVN}\left( \mathbf{G}_{\mathbf{jk}}\boldsymbol{\beta}_{\mathbf{k}}+\mathbf{Z}_{\mathbf{ik}}\boldsymbol{\gamma}_{\mathbf{k}},\sigma^{2}\mathbf{I} \right)$.

As in Methods, we estimate the partial covariance between genotypes and phenotypes using the meta-analysis score statistics:

$\hat{\rho}_{GY|Z,j}=\frac{\sum_{k\in\left\{ k:M_{jk}=1 \right\}} U_{jk}}{\sum_{k\in\left\{ k:M_{jk}=1 \right\}} N_{jk}}$

$\hat{\rho}_{GG|Z,j_{1}j_{2}}=\frac{\sum_{k\in\left\{ k:M_{j_{1}k}=M_{j_{2}k}=1 \right\}} V_{j_{1}j_{2}k}}{\sum_{k\in\left\{ k:M_{j_{1}k}=M_{j_{2}k}=1 \right\}} N_{jk}}$ (S3)

The expectation for the score statistic $U_{jk}$ is equal to

$$E\left( U_{jk} \right)=\frac{1}{\hat{\sigma}_{k}^{2}}\mathbf{G}_{\mathbf{jk}}^{\mathbf{T}}\left( \mathbf{I}-\left( \mathbf{Z}_{\mathbf{k}}^{\mathbf{T}}\mathbf{Z}_{\mathbf{k}} \right)^{-1}\mathbf{Z}_{\mathbf{k}}^{\mathbf{T}} \right)\mathbf{G}_{\mathbf{k}}\boldsymbol{\beta}$$

Therefore, the mean values for the partial covariance estimator are equal to

$$E\left( \hat{\rho}_{GY|Z,j} \right)\sim\frac{1}{\hat{\sigma}_{k}^{2}}\sum_{j^{'}} \sum_{k\in\left\{ k:M_{jk}=M_{j^{'}k}=1 \right\}} \frac{\mathbf{G}_{\mathbf{jk}}^{\mathbf{T}}\left( \mathbf{I}-\left( \mathbf{Z}_{\mathbf{k}}^{\mathbf{T}}\mathbf{Z}_{\mathbf{k}} \right)^{-1}\mathbf{Z}_{\mathbf{k}}^{\mathbf{T}} \right)\mathbf{G}_{\mathbf{j}^{\mathbf{'}}\mathbf{k}}\beta_{j^{'}}}{\sum_{k\in\left\{ k:M_{jk}=M_{j^{'}k}=1 \right\}} N_{k}}\sim\sum_{j^{'}=1}^{J} \hat{\rho}_{GG|Z,jj^{'}}\beta_{j}^{'}$$

Therefore, the joint genetic effect estimate using the partial variance-covariance matrix is consistent, i.e.

$$E\left[ {\hat{\boldsymbol{\rho}}}_{\mathbf{GG|Z}}^{\boldsymbol{-1}}{\hat{\boldsymbol{\rho}}}_{\mathbf{GY|Z}} \right]\boldsymbol{=}\boldsymbol{\beta}$$

**3. Conditional Meta-analysis of Gene-level Association Tests with Partial Correlation Based Score Statistics**

As shown by us and others, commonly used gene-level tests (e.g. burden test [2], sequence kernel association test [3] and variable threshold test [4]) can be calculated using vectors of signal variant association statistics and their covariance matrices. After conditional partial correlation based score statistics are obtained, gene-level tests can be constructed in the same way to distinguish independently associated genes from shadows of known associations. Specifically, the burden test statistic and its variance can be calculated by

$U_{BURDEN}=\mathbf{w}^{\mathbf{T}}{\tilde{\mathbf{U}}}_{\mathbf{G|}\mathbf{G}^{\mathbf{*}}}$ and $V_{BURDEN}=\mathbf{w}^{\mathbf{T}}{\tilde{\mathbf{V}}}_{\mathbf{G|}\mathbf{G}^{\mathbf{*}}}\mathbf{w}$ (S4)

where $\mathbf{w}$ is the weights assigned to each variant. The standardized burden statistic satisfies $T_{BURDEN}=\frac{U_{BURDEN}}{V_{BURDEN}^{1/2}}\sim N\left( 0,1 \right)$. The SKAT statistic[3] is equal to

$Q_{SKAT}={\tilde{\mathbf{U}}}_{\mathbf{G|}\mathbf{G}^{\mathbf{*}}}^{\mathbf{T}}\boldsymbol{\Omega} {\tilde{\mathbf{U}}}_{\mathbf{G|}\mathbf{G}^{\mathbf{*}}}$ (S5)

where $\boldsymbol{\Omega}$ contains the weights assigned to each variant site. The SKAT statistic follows a mixture chi-square distribution with mixture proportions being the eigenvalues for $\mathbf{V}_{\mathbf{GENE|KNOWN}}^{*1/2}\boldsymbol{\Omega}\mathbf{V}_{\mathbf{GENE|KNOWN}}^{\mathbf{*1/2}}$. The VT statistic [4] calculates a burden test statistic for each minor allele frequency threshold, and corrects for the multiple comparison using the minimal p-value method. The p-values can be calculated using the cumulative distribution function for the multivariate normal distribution [5].

**4. Description of the Participating Cohorts In Meta-analysis of Nicotine Addiction Phenotype**

We describe below the study design and analysis protocols for the studies used in the meta-analysis of cigarettes per day phenotype.

SardiNIA study on aging (SardiNIA)

The SardiNIA study is a longitudinal, population-based study that includes 6,921 individuals, representing >60% of the adult population of 4 villages in the Lanusei valley on Sardinia (Italy)[6]. These individuals are clustered in 1,257 multigenerational families, up to 5 generations deep, and have been characterized for hundreds of quantitative traits. All participants gave informed consent to study protocols, which were approved by the Sardinian local research ethic committees: Comitato Etico di Azienda Sanitaria Locale 8, Lanusei (2009/0016600) and Comitato Etico di Azienda Sanitaria Locale 1, Sassari (2171/CE)) and by the NIH Office of Human Subjects Research as governed by Italian institutional review board approval. In association analysis, the covariates age, age^2, sex and status of former smoker were adjusted. A linear mixed model was used to perform the association analysis.

Metabolic Syndrome in Men Study (METSIM)

The METSIM study aims to investigate the metabolic syndrome, type 2 diabetes, cardiovascular disease, and cardiovascular risk factors[7]. It is an ongoing study of men aged 50 to 70 years, randomly selected from the population registry of the town of Kuopio, in Eastern Finland. In association analysis, the covariates age, age^2 and sex were adjusted. A linear mixed model was used to perform the association analysis.

Minnesota Center for Twin and Family Research (MCTFR).

The MCTFR sample is composed of two primary cohorts, a population-based sample of twins and their parents, and a sample of families with adopted children. The study design and genetic data have been described in detail in prior publications[7-10]. In association analysis, the covariates age, age^2 and sex were adjusted. A linear mixed model was used to perform the association analysis.

Center for Antisocial Drug Dependence (CADD)

The CADD is a study of over 8000 individuals from which participants were selected for genotyping [11]. Participants were selected for genotyping based on a measure of behavioral disinhibition taken in adolescence. Complete information on the genotyped sample and selection procedure have been described in detail previously[12]. In association analysis, the covariates age, age^2 and sex were adjusted. A linear mixed model was used to perform the association analysis.

Genes for Good

Genes for Good is an online study of the genetics of health and behavior (https://apps.facebook.com/genesforgood). Participation in Genes for Good is open to anyone over age 18 with a U.S. postal address. The covariates of age, age^2, sex, weight, height and 20 top principal components were adjusted. A linear mixed model was used to perform the association analysis.

COPDGene

COPDgene is a multisite observational study designed to research genetic factors that affect chronic obstructive pulmonary disease (COPD). Detailed information can be found in prior publications.[13] Individuals of European ancestry was analyzed. The covariates of age, age^2 and sex were adjusted. A linear mixed model was used to perform the association analysis.

UK Biobank Dataset

The full UK Biobank dataset is composed of ~500,000 individuals with detailed measurement of smoking behaviors and other health outcomes. The samples were either genotyped via UK BiLEVE Affymetrix array or the Affymetrix Axiom array. The genetic data were phased and imputed to the Haplotype Consortium Reference panel. Comprehensive detail on the UK Biobank can be found here: <http://www.ukbiobank.ac.uk/> .

**REFERENCES**

1. Qiao D, Lange C, Beaty TH, Crapo JD, Barnes KC, Bamshad M, et al. Exome Sequencing Analysis in Severe, Early-Onset Chronic Obstructive Pulmonary Disease. Am J Respir Crit Care Med. 2016;193(12):1353-63. doi: 10.1164/rccm.201506-1223OC. PubMed PMID: 26736064.

2. Li B, Leal SM. Methods for detecting associations with rare variants for common diseases: application to analysis of sequence data. American journal of human genetics. 2008;83(3):311-21. Epub 2008/08/12. doi: 10.1016/j.ajhg.2008.06.024. PubMed PMID: 18691683; PubMed Central PMCID: PMC2842185.

3. Wu MC, Lee S, Cai T, Li Y, Boehnke M, Lin X. Rare-variant association testing for sequencing data with the sequence kernel association test. American journal of human genetics. 2011;89(1):82-93. Epub 2011/07/09. doi: S0002-9297(11)00222-9 [pii]

10.1016/j.ajhg.2011.05.029. PubMed PMID: 21737059.

4. Price AL, Kryukov GV, de Bakker PI, Purcell SM, Staples J, Wei LJ, et al. Pooled association tests for rare variants in exon-resequencing studies. American journal of human genetics. 2010;86(6):832-8. Epub 2010/05/18. doi: S0002-9297(10)00207-7 [pii]

10.1016/j.ajhg.2010.04.005. PubMed PMID: 20471002.

5. Lin DY, Tang ZZ. A general framework for detecting disease associations with rare variants in sequencing studies. American journal of human genetics. 2011;89(3):354-67. doi: 10.1016/j.ajhg.2011.07.015.

10.1016/j.ajhg.2011.07.015. PubMed PMID: 21885029; PubMed Central PMCID: PMC3169821.

6. Pilia G, Chen WM, Scuteri A, Orru M, Albai G, Dei M, et al. Heritability of cardiovascular and personality traits in 6,148 Sardinians. PLoS genetics. 2006;2(8):e132. doi: 10.1371/journal.pgen.0020132. PubMed PMID: 16934002; PubMed Central PMCID: PMCPMC1557782.

7. Stancakova A, Javorsky M, Kuulasmaa T, Haffner SM, Kuusisto J, Laakso M. Changes in insulin sensitivity and insulin release in relation to glycemia and glucose tolerance in 6,414 Finnish men. Diabetes. 2009;58(5):1212-21. doi: 10.2337/db08-1607. PubMed PMID: 19223598; PubMed Central PMCID: PMCPMC2671053.

8. Miller MB, Basu S, Cunningham J, Eskin E, Malone SM, Oetting WS, et al. The Minnesota Center for Twin and Family Research genome-wide association study. Twin Research and Human Genetics. 2012;15(6):767-74.

9. Iacono WG, McGue M, Krueger RF. Minnesota Center for Twin and Family Research. Twin Research and Human Genetics. 2006;9(6):978-84. Epub 2007/01/27. doi: 10.1375/183242706779462642. PubMed PMID: 17254440.

10. Iacono WG, McGue M. Minnesota Twin Family Study. Twin Res. 2002;5(5):482-7. PubMed PMID: 12537881.

11. Stallings MC, Corley RP, Dennehey B, Hewitt JK, Krauter KS, Lessem JM, et al. A genome-wide search for quantitative trait Loci that influence antisocial drug dependence in adolescence. Arch Gen Psychiatry. 2005;62(9):1042-51. doi: 10.1001/archpsyc.62.9.1042. PubMed PMID: 16143736.

12. Derringer J, Corley RP, Haberstick BC, Young SE, Demmitt BA, Howrigan DP, et al. Genome-Wide Association Study of Behavioral Disinhibition in a Selected Adolescent Sample. Behav Genet. 2015;45(4):375-81. doi: 10.1007/s10519-015-9705-y. PubMed PMID: 25637581; PubMed Central PMCID: PMCPMC4459903.

13. Regan EA, Hokanson JE, Murphy JR, Make B, Lynch DA, Beaty TH, et al. Genetic epidemiology of COPD (COPDGene) study design. COPD. 2010;7(1):32-43. doi: 10.3109/15412550903499522. PubMed PMID: 20214461; PubMed Central PMCID: PMCPMC2924193.
