# Supplementary figures and images for: Proper conditional analysis in the presence of missing data: Application to large scale meta-analysis of tobacco use phenotypes
Source: PLoS Genet. 2018 Jul 17;14(7):e1007452. doi: 10.1371/journal.pgen.1007452 (PMC6063450; doi:10.1371/journal.pgen.1007452)

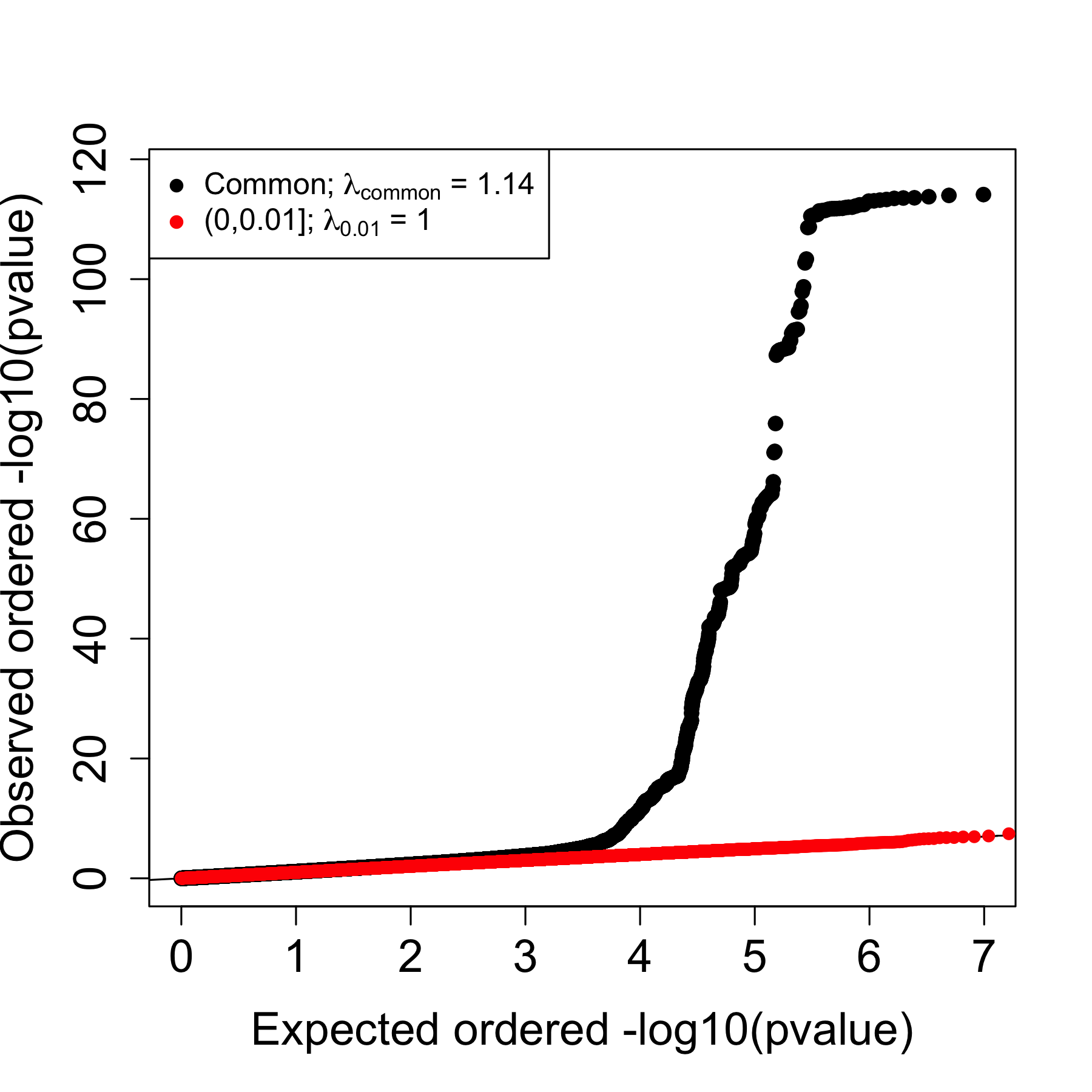

Supplement: S1 Fig — Genomic control values were separately reported for variants with MAF>0.01 and with MAF<0.01. (TIFF) [file pgen.1007452.s002.tiff]

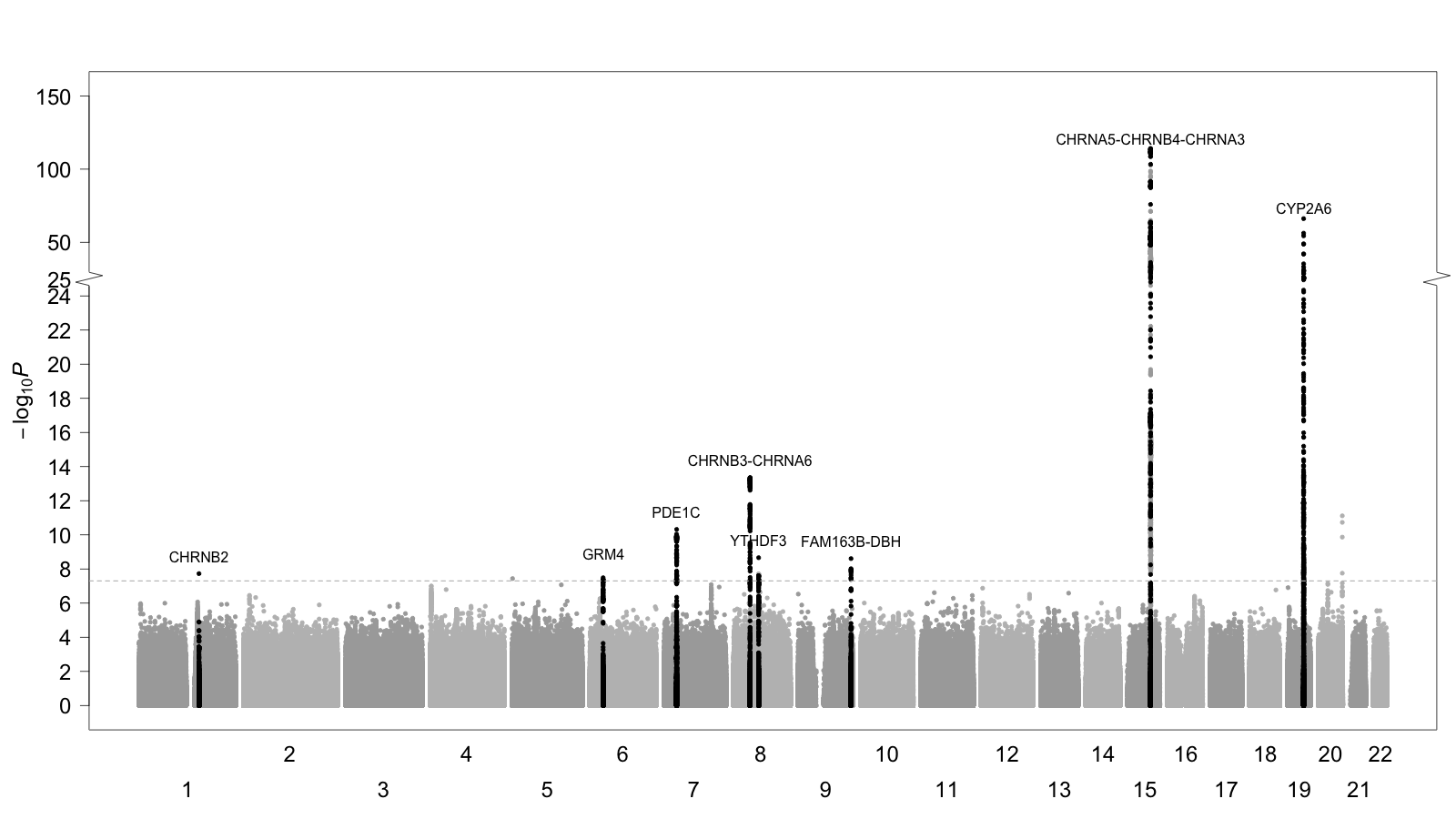

Supplement: S2 Fig — (TIFF) [file pgen.1007452.s003.tiff]
