# Supplementary material for: Proper conditional analysis in the presence of missing data: Application to large scale meta-analysis of tobacco use phenotypes
Source: PLoS Genet. 2018 Jul 17;14(7):e1007452. doi: 10.1371/journal.pgen.1007452 (PMC6063450; doi:10.1371/journal.pgen.1007452)
Supplement: S1 Table — We evaluated the impact of large heterogeneity in the genetic effects on the power and type I errors for the PCBS statistics. The effects of the conditioned variants in each cohort are sampled from the distribution N(μβ2,(μβ2/2)2). All other simulation settings are the same as in Table 1. (DOCX) [file pgen.1007452.s004.docx]

**S1 Table**: **Power and Type I Errors of Meta-analysis of Single Variant Tests in the Presence of Missing Data and Genetic Effect Heterogeneity.** We evaluated the impact of large genetic effect heterogeneity on the power and type I errors for the partial correlation based score statistics. The effects of the conditioned variants in each cohort are sampled from the distribution $N\left( \mu_{\beta_{G^{*}}},{{(\mu}_{\beta_{G^{*}}}/2)}^{2} \right)$. All other simulation settings are the same as in Table 1.

| Conditioned Variant  Effect | Candidate Variant Effect | Fraction of  Missing Data | Type I Error/Power | | | | | | |
| --- | --- | --- | --- | --- | --- | --- | --- | --- | --- |
|  |  |  | PCBS | Synthesis Analysis | ImpG- Summary | COJO | DISCARD | REPLACE0 | Analyze the Full Dataset [Gold Standard] |
|  |  |  | Type I Error | | | | | | |
| 0.04 | 0 | 0.1 | $5.1\times{10}^{-3}$ | $3.8\times{10}^{-3}$ | 0.38 | $0.012$ | $4.1\times{10}^{-3}$ | $7.4\times{10}^{-3}$ | $5.1\times{10}^{-3}$ |
| 0.04 | 0 | 0.3 | $5.6\times{10}^{-3}$ | $4.3\times{10}^{-3}$ | $0.89$ | 0.036 | $3.9\times{10}^{-3}$ | $0.064$ | $5.2\times{10}^{-3}$ |
| 0.04 | 0 | 0.5 | $5.8\times{10}^{-3}$ | $4.3\times{10}^{-3}$ | $0.99$ | 0.050 | $2.2\times{10}^{-3}$ | $0.20$ | $4.9\times{10}^{-3}$ |
| 0.08 | 0 | 0.1 | $5.5\times{10}^{-3}$ | $2.2\times{10}^{-3}$ | 0.65 | $0.034$ | $2.2\times{10}^{-3}$ | $0.015$ | $5.0\times{10}^{-3}$ |
| 0.08 | 0 | 0.3 | $5.7\times{10}^{-3}$ | $1.8\times{10}^{-3}$ | $0.97$ | 0.077 | $2.1\times{10}^{-3}$ | $0.22$ | $5.2\times{10}^{-3}$ |
| 0.08 | 0 | 0.5 | $5.2\times{10}^{-3}$ | $1.3\times{10}^{-3}$ | $0.25$ | 0.65 | $9.3\times{10}^{-4}$ | 0.60 | $4.9\times{10}^{-3}$ |
|  |  |  | Power | | | | | | |
| 0.04 | 0.04 | 0.1 | 0.26 | 0.24 | - | - | 0.14 | - | 0.28 |
| 0.04 | 0.04 | 0.3 | 0.20 | 0.19 | - | - | 0.028 | - |  |
| 0.04 | 0.04 | 0.5 | 0.15 | 0.14 | - | - | $5.7\times{10}^{-3}$ | - |  |
| 0.08 | 0.04 | 0.1 | 0.26 | 0.20 | - | - | $0.11$ | - | 0.29 |
| 0.08 | 0.04 | 0.3 | 0.21 | 0.15 | - | - | 0.019 | - |  |
| 0.08 | 0.04 | 0.5 | 0.14 | 0.11 | - | - | $3.6\times{10}^{-3}$ | - |  |
| 0.04 | 0.08 | 0.1 | 0.68 | 0.67 | - | - | 0.53 | - | 0.70 |
| 0.04 | 0.08 | 0.3 | 0.61 | 0.60 | - | - | 0.16 | - |  |
| 0.04 | 0.08 | 0.5 | 0.53 | 0.53 | - | - | 0.024 | - |  |
| 0.08 | 0.08 | 0.1 | 0.68 | 0.63 | - | - | 0.48 | - | 0.70 |
| 0.08 | 0.08 | 0.3 | 0.61 | 0.56 | - | - | 0.13 | - |  |
| 0.08 | 0.08 | 0.5 | 0.50 | 0.48 | - | - | $0.017$ | - |  |
