# Supplementary material for: Proper conditional analysis in the presence of missing data: Application to large scale meta-analysis of tobacco use phenotypes
Source: PLoS Genet. 2018 Jul 17;14(7):e1007452. doi: 10.1371/journal.pgen.1007452 (PMC6063450; doi:10.1371/journal.pgen.1007452)
Supplement: S2 Table — We evaluated the impact of large heterogeneity in the genetic effects on the power and type I errors for the PCBS statistics. The genetic effects for the conditioned variants in each cohort are sampled from the distribution N(μβ2,(μβ2/2)2). All other simulation settings are the same as in Table 2. (DOCX) [file pgen.1007452.s005.docx]

**S2 Table: Power and Type I Errors of Meta-analysis of Gene-level Tests in the Presence of Missing Data and Genetic Effect Heterogeneity.** We evaluated the impact of large genetic effect heterogeneity on the power and type I errors for the partial correlation based score statistics. The genetic effects for the conditioned variants in each cohort are sampled from the distribution $N\left( \mu_{\beta_{G^{*}}},\left( \frac{\mu_{\beta_{G^{*}}}}{2} \right)^{2} \right)$. All other simulation settings are the same as in Table 2.

| **Mean Conditioned Variant Effect** | **Candidate Variant Effect**  **(**$\boldsymbol{\tau}_{\boldsymbol{\beta}}$**)** | **Fraction of Missing Data** | **Type I Error/Power for Burden/SKAT/VT (α=0.0005)** | | |
| --- | --- | --- | --- | --- | --- |
|  |  |  | **Partial Correlation Based Score Statistics** | **Analyze the Full Dataset**  **[Gold Standard]** |  |
| 0.05 | 0 | 0.1 | 3.4$\times{10}^{-4}$/3.4$\times{10}^{-4}$/3.4$\times{10}^{-4}$ | 3.8$\times{10}^{-4}$/4.5$\times{10}^{-4}$/4.5$\times{10}^{-4}$ |  |
| 0.05 | 0 | 0.3 | 4.0$\times{10}^{-4}$/3.7$\times{10}^{-4}$/5.4$\times{10}^{-4}$ | 4.4$\times{10}^{-4}$/4.7$\times{10}^{-4}$/6.7$\times{10}^{-4}$ |  |
| 0.05 | 0 | 0.5 | 6.5$\times{10}^{-4}$/5.5$\times{10}^{-4}$/4.6$\times{10}^{-4}$ | 6.5$\times{10}^{-4}$/6.5$\times{10}^{-4}$/5.2$\times{10}^{-4}$ |  |
| 0.1 | 0 | 0.1 | 6.0$\times{10}^{-4}$/4.2$\times{10}^{-4}$/5.3$\times{10}^{-4}$ | 5.6$\times{10}^{-4}$/5.6$\times{10}^{-4}$/6.0$\times{10}^{-4}$ |  |
| 0.1 | 0 | 0.3 | 5.4$\times{10}^{-4}$/4.7$\times{10}^{-4}$/4.7$\times{10}^{-4}$ | 5.8$\times{10}^{-4}$/5.8$\times{10}^{-4}$/5.4$\times{10}^{-4}$ |  |
| 0.1 | 0 | 0.5 | 4.5$\times{10}^{-4}$/6.8$\times{10}^{-4}$/5.8$\times{10}^{-4}$ | 5.8$\times{10}^{-4}$/6.8$\times{10}^{-4}$/4.9$\times{10}^{-4}$ |  |
| 0.05 | 0.1 | 0.1 | 0.21/0.21/0.19 | 0.22/0.23/0.21 |  |
| 0.05 | 0.1 | 0.3 | 0.19/0.19/0.17 |  |  |
| 0.05 | 0.1 | 0.5 | 0.18/0.16/0.15 |  |  |
| 0.1 | 0.1 | 0.1 | 0.22/0.22/0.20 | 0.22/0.23/0.21 |  |
| 0.1 | 0.1 | 0.3 | 0.20/0.20/0.18 |  |  |
| 0.1 | 0.1 | 0.5 | 0.17/0.16/0.15 |  |  |
| 0.05 | 0.2 | 0.1 | 0.59/0.59/0.58 | 0.60/0.61/0.59 |  |
| 0.05 | 0.2 | 0.3 | 0.56/0.56/0.54 |  |  |
| 0.05 | 0.2 | 0.5 | 0.54/0.53/0.52 |  |  |
| 0.1 | 0.2 | 0.1 | 0.59/0.59/0.57 | 0.59/0.60/0.59 |  |
| 0.1 | 0.2 | 0.3 | 0.57/0.57/0.55 |  |  |
| 0.1 | 0.2 | 0.5 | 0.55/0.54/0.52 |  |  |
